# Mean Carotid Intima-Media Thickness in Patients with Type 2 Diabetes Mellitus Attending Tertiary Care Center: A Descriptive Cross-sectional Study

**DOI:** 10.31729/jnma.7136

**Published:** 2021-12-31

**Authors:** Prakash Kayastha, Sharma Paudel, Ghanshyam Gurung, Pradeep Kumar, Rudra Prasad Upadhyaya, Sasmita Tuladhar, Govinda Adhikari, Santosh Maharjan

**Affiliations:** 1 Department of Radiology and Imaging, Maharajgunj Medical Campus, Tribhuvan University Teaching Hospital, Institute of Medicine, Maharajgunj, Kathmandu, Nepal; 2 Department of Radiology and Imaging, Grande International Hospital, Dhapasi, Kathmandu, Nepal; 3 Department of Radiology and Imaging, Kanti Children's Hospital, Maharajgunj, Kathmandu, Nepal

**Keywords:** *atherosclerosis*, *carotid intima media thickness*, *diabetes mellitus type 2*

## Abstract

**Introduction:**

Sonographic carotid intima media thickness measurement in diabetic patients is an important tool for estimating the risk of cardiovascular and cerebrovascular events. It is a simple, noninvasive and widely available tool which can give idea of further treatment needed. The objective of this study was to determine the mean intima media complex thickness in patients with type II diabetes mellitus attending a tertiary care center.

**Methods:**

This was a descriptive cross-sectional study done in 64 patients with the type II diabetes mellitus patients visiting diabetic clinic of Tribhuvan University Teaching Hospital, sent for carotid Doppler examination in the department of radiology and imaging. Ethical approval was taken from the Instituitonal Review Board. Convenient sampling method was used. Carotid intima media thickness was measured on both sides and mean intima media thickness was calculated. Mean intima media thickness for male and female diabetic patients was also calculated separately. Statistical Package for Social Sciences version 25 was used for data analysis.

**Results:**

The mean carotid intima media thickness was 0.86±0.13mm with range from 0.7mm to 1.3mm. Mean intima media thickness in male was 0.832±0.094mm and in female it was 0.904±0.144mm. Among 64 patients, 30 (46.8%) were female and 34 (53.3%) were male. Age of the patients ranged from 35 years to 68 years with mean age of 52.4±6.54years.

**Conclusions:**

Carotid intima media thickness in patients with type II diabetes mellitus showed higher values than that of mean value from study done in similar study. Female had higher mean intima media thickness than male.

## INTRODUCTION

Sonographic evaluation of carotid artery intimamedia thickness (CIMT) is a simple, noninvasive, widely available and reproducible imaging parameter. Intima-media thickness (IMT) is an early marker of atherosclerosis, independent predictor of cardiovascular and cerebrovascular disease and also used for effectiveness of medical therapies in treating atherosclerosis. Noninvasive techniques such as B-mode ultrasound can directly assess the IMT, which corresponds to the thickness of the histologic intima and media.^1-3^

Diabetes mellitus (DM) is considered a disease which accelerates atherosclerotic changes. IMT is considered to correlate well with the duration of diabetes and various parameters associated with it.^4-7^

Patients with type-2 diabetes mellitus have greater carotid intima media thickness.^8^ IMT measurement should be included as a diagnostic tool, given the paucity of facilities for invasive techniques in Nepal. This may help in early identification of coronary artery disease.

The aim of this study was to find out the mean CIMT in type II diabetic patients attending a tertiary care center.

## METHODS

This was a descriptive cross-sectional study done in patients with type 2 Diabetes mellitus visiting the diabetic clinic of Tribhuvan University Teaching Hospital (TUTH) and patients visiting the Department of Radiology and Imaging for carotid Doppler examination.

An ethical clearance had been obtained from the institutional review committee of the Institute of Medicine, Maharajgunj. The study duration was one year from September 2019 to September 2020. Convenient sampling method was used. Patients with Type 2 Diabetes Mellitus (both newly diagnosed and old cases), patients aged more than 25 years and both sexes were included in the study. Type 1 diabetic, uncontrolled type 2 diabetic and patient <25 years were not included in this study.

Sample size was calculated by using following formula:

n = Z^2^ × σ^2^ / e^2^

  = (1.64)^2^ × (0.5)^2^/ (0.11)^2^

  = 56

Where

n = minimum number of sample required
Z= 1.64 at 90% Confidence Interval (CI)
σ = standard deviation taken from the previous study
e = margin of error, 11%

From the above mentioned formula minimum sample required for the study was calculated to be 56. We enrolled 64 patients in our study. Convenient sampling method was used to collect sample.

The common and internal carotid arteries of both sides were evaluated sonographically, using high frequency linear probe (7-12MHz) of Samsung Medison Accuvix A30 ultrasound machine, with the patient in supine position and the examiner seated near the patient's head. The patient's head was tilted away from the side being examined facilitating the neck exposure. With this technique, two parallel echogenic lines separated by an anechoic space can be visualized at the artery wall. These lines are generated by the blood-intima and media-adventitial interfaces. The distance between the two lines gives a reliable index of the thickness of the intimal-media complex.^9^ The IMT of the common and internal carotid arteries was measured within one cm of the carotid bulb. IMT of only the plaque-free segments was recorded.

A total of eight IMT values were taken from different sites including the near and far walls of common and internal carotid arteries on both sides: the near and far wall of the proximal 8mm of the internal carotid artery, the near and far wall of the carotid bifurcation beginning at the tip of the flow divider and extending 8 mm proximally, and the near and far wall of the arterial segment extending 8 to 16mm proximally to the tip of the flow divider into the common carotid artery.^10^ Average values of these measurements for each participant was calculated to find out the mean IMT.

The mean value of the above was obtained and designated as the mean IMT value of that subject as follows:

(RCN: Near wall of Right CCA, RCF: Far wall of right CCA, RIN: Near wall of right ICA, RIF: Far wall of right ICA, LCN: Near wall of left CCA, LCF: Far wall of left CCA, LIN: Near wall of left ICA, LIF: Far wall of left ICA).

Data was entered in Microsoft excel and analysis was carried out using Statistical Package for Social Sciences (SPSS) version 25 and mean values for the numerical variables were calculated.

## RESULTS

The mean intima media thickness is 0.86 ±0.12mm. The mean IMT values ranged from 0.70mm to 1.3mm with median of 0.85mm and. Mean IMT value in male patients was found to be 0.832±0.094mm and in female it was found to be 0.904±0.144mm. The values of IMT in females were higher than those of males.

Out of the 64 patients, 30 (46.8%) were female and 34 (53.3%) were males (Table 1).

**Table 1:** Gender wise distribution.

Gender	n (%)
Male	34 (53.30)
Female	30 (46.80)

**Figure 1:**
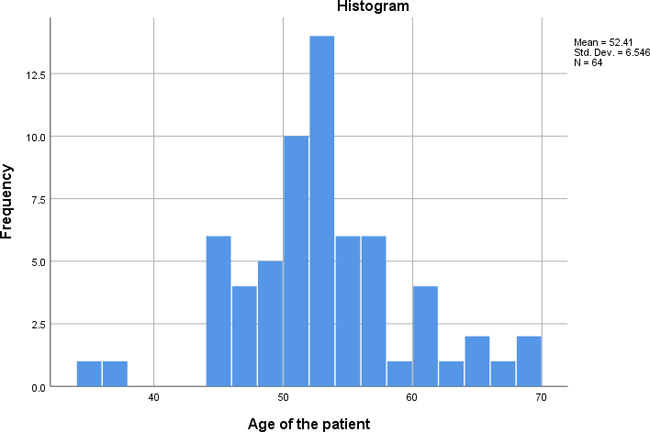
Histogram showing age distribution of patients.

The age of the participants ranged from 35 years to 68 years with mean age of 52.41, median age of 52 years (variance 42.848, standard deviation 6.546) (Figure 1).

## DISCUSSION

In this study we measured carotid intima media thickness in 64 type II diabetes patients. The mean thickness was 0.86±0.12mm. Mean carotid intima media thickness from this study in type II diabetes patients was higher than that of mean thickness from the study in normal population by Paul J, et al. and Randrianarisoa E, et al.^11,12^ Increase in carotid intima media thickness in patients with type II diabetes is shown in study done by Kota SK, et al. and Paul J, et al. which is consistent with this study.^13,14^ The rate of change in IMT with age has been calculated to be about 0.01mm/year in the general population and 0.03-0.06mm/year in patients with coronary artery disease.^15^ Type II diabetes is considered as a risk factor for coronal artery disease and ultrasonography as a reliable and accurate technique to determine IMT in the superficial arteries. Thus, carotid IMT measurement and peripheral plaque detection by using B mode ultrasound is of clinical value in the screening of patients with coronary artery disease.^1,16,17^

Mean IMT in this study showed higher values in female than that of male which was similar to that of study that was carried out by Zakaria M, et al. Mean IMT in females was higher than that in males in the study (0.90mm in males and 0.91 in females).^7^ However studies carried out by Baba, et al. showed similar intima media thickness values in male and female population.^13^ While other studies carried out by Pujia A, et al. and Theodora S, et al. showed male patients to have higher intima media thickness than females.^14,15^ This discrepancy in literature requires further investigations on this field.

Few limitations of the study are worth mentioning, Measurements were difficult in obese and short necked individuals, especially for measuring the intima media thickness of internal carotid arteries. There was difficulty in visualizing the intima media thickness due to near field artifacts in some cases.

## CONCLUSIONS

Carotid intima media thickness measured in patients with type II diabetes mellitus were higher than that of mean value from study done in similar study. Increased intima media thickness is considered a risk factor for cardiovascular and cerebrovascular events. Examination with ultrasound which is a safe, non-invasive and widely available tool, can predict the occurrence of these events so that they can be prevented by timely intervention. From the study, routine and timely evaluation of carotid intima-media thickness is strongly recommended.
